# 1308. Exploration of Infectious Disease Specialist Attitudes on Utilizing Oral Antimicrobials for the Treatment of Bone and Joint Infections

**DOI:** 10.1093/ofid/ofad500.1147

**Published:** 2023-11-27

**Authors:** Katy Wolfe, Jason Funaro, Jessica Seidelman, Kristen Dicks, Michael E Yarrington, Jenny Shroba

**Affiliations:** Duke University Hospital, Durham, North Carolina; Duke University Hospital, Durham, North Carolina; Duke University School of Medicine, Durham, NC; Duke University Health System, Durham, North Carolina; Duke University Health System, Durham, North Carolina; Duke University Hospital, Durham, North Carolina

## Abstract

**Background:**

Despite recent literature supporting non-inferiority of oral therapy, many patients are treated with traditional extended intravenous (IV) antimicrobial courses. The objective of this study was to evaluate the attitudes of infectious disease (ID) specialists on utilizing oral antimicrobials (PO-A) for the treatment of bone and joint infections (BJI).

**Methods:**

This was a multicenter clinician survey study of ID specialists from 1/10/23 – 1/31/23, regarding their attitude towards using PO-A for the treatment of BJIs. Survey respondents were presented with statements and rank order questions and asked to indicate their level of agreeance using a 5-point Likert scale. The primary outcome was agreement with the phrase ‘I am willing to prescribe PO-A for treatment of BJIs’. Secondary outcomes included patient case and ID specialist characteristics associated with willingness to prescribe PO-A. Study utilized descriptive statistics and univariate tests.

**Results:**

A total of 216 surveys were distributed to ID specialists of which 65 recorded responses with 50 complete responses (30% overall response rate and 23% completion rate). Of the 50 complete surveys, the majority of respondents (60%) indicated they prescribe PO-A in ≤25% of their patient-case load as definitive treatment of BJIs. A total of 41/50 respondents (82%) indicated strong agreement with comfortability prescribing and dosing PO-A for BJIs (Table 1) and were most willing to prescribe PO-A ( >70%) in patients with diabetic foot infection (Table 2). ID specialists’ willingness to prescribe PO-A for treatment of BJIs significantly correlated with their current clinical practice. Respondents who disagreed with the statement ‘I am willing to prescribe PO-A’ currently prescribe PO-A in less than 25% of their BJI patients compared to respondents who agreed (37% prescribe PO-A 26-50% of their patients with 35% of those who strongly agree prescribe PO-A it in >50% of their BJI patients (p=0.004). The most common concern (62%) when prescribing PO-A for BJIs is the identified pathogen may not be susceptible to the preferred antibiotic (Table 3).

**Figure:**
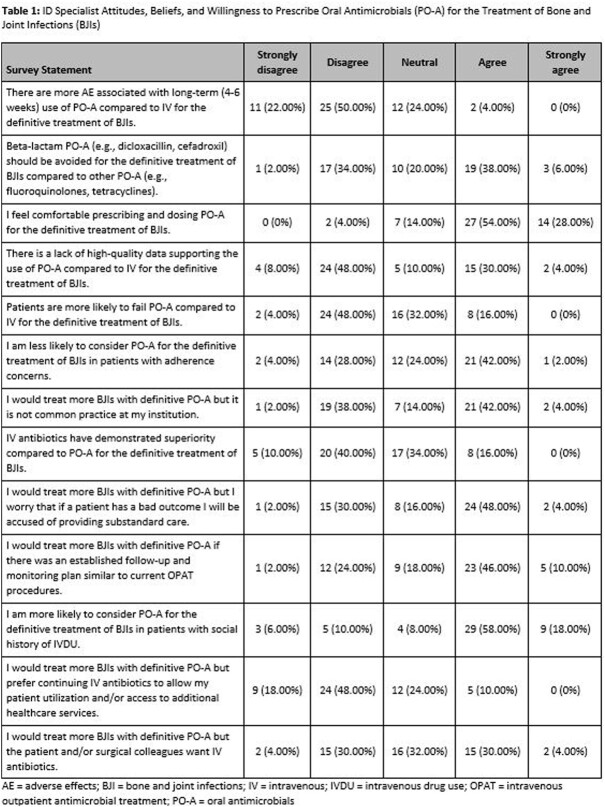

**Figure:**
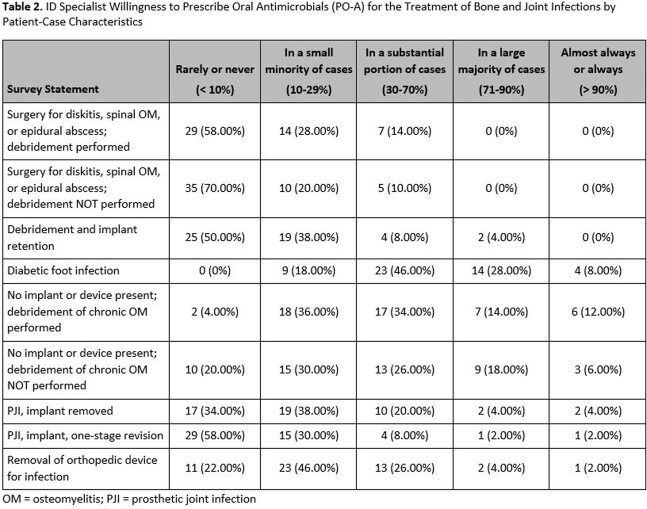

**Figure:**
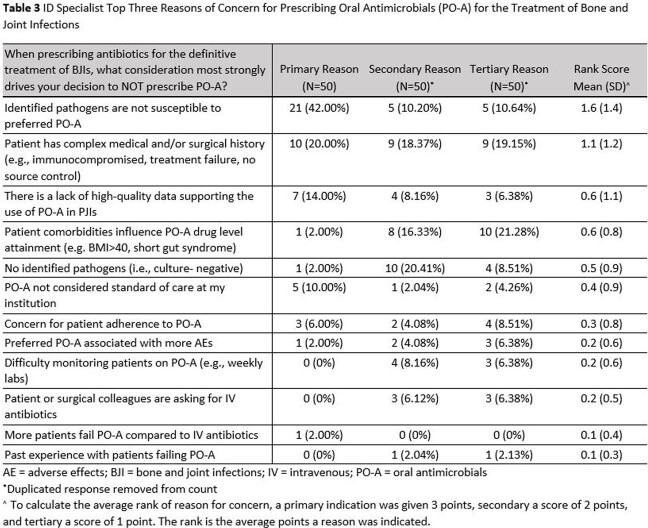

**Conclusion:**

The findings of this survey suggest that most ID specialists in this sample are comfortable prescribing PO-A for treatment of BJIs and was significantly related to current practice.

**Disclosures:**

**Jessica Seidelman, MD, MPH**, Uptodate: content editor for pelvic osteomyelitis page **Kristen Dicks, MD**, UpToDate: Advisor/Consultant

